# Contrasting diversity and temporal patterns in leaf and root microbiome of two nearby temperate* Zostera marina* meadows

**DOI:** 10.1186/s40793-025-00760-z

**Published:** 2025-08-05

**Authors:** E. Teira, A. Díaz-Alonso, E. Fernández, M. Pérez-Lorenzo, E. Delgadillo-Nuño, C. Mendoza-Segura, J. Severino P. Ibánhez, P. Beca-Carretero

**Affiliations:** 1 Departamento de Ecoloxía E Bioloxía Animal, Centro de Investigación Mariña, Universidade de Vigo, Vigo, Spain; 2 Centro Oceanografico de A Coruña, Instituto Español de Oceanografia-CSIC, A Coruña, Spain; 3 Laboratorio de Geoquímica Orgánica, IIM-CSIC, Vigo, Spain

**Keywords:** *Zostera marina*, Leaf and root microbiome, Diversity, Succession, Nitrogen and sulphur cycles

## Abstract

**Background:**

The *Zostera marina* microbiome plays a crucial role in meadow functioning and resilience. We aim at testing if the microbial communities of *Z. marina* leaves and roots exhibit distinct diversity and succession patterns associated to distinct environmental conditions and anthropogenic pressures. Site-specific and temporal changes of *Z. marina* leaf and root microbiomes were assessed in the urban beach of Bouzas and the rural beach of Cesantes in NW Spain from March 2021 to March 2022.

**Results:**

The prokaryotic microbiome from leaves significantly differed from that in roots, and 33% of the OTUs were shared by both tissues. Significant differences in taxonomic composition were found between Cesantes and Bouzas, yet about half of the taxa were common to both locations, suggesting a host-specific core microbiome. Prokaryote diversity in roots was significantly higher than in leaves, and significantly higher in Bouzas than in Cesantes, while the diversity in leaves was higher in Cesantes. In *Z. marina* leaves, the dominant order *Granulosicoccales* was more abundant in Bouzas than in Cesantes, which could be indicative of anthropogenic pressures. *Desulfobacterota* was the dominant microbial group in roots, especially in summer. Many microbial taxa associated to the roots were positively correlated with plant growth, suggesting a positive effect of root microbiome on the plant. An apparent succession pattern was observed in the leaf and, to a lesser extent, root microbiomes in Bouzas, with communities from the beginning of the growing season (March) strongly resembling between the two sampling years. By contrast, leaf and root microbiomes in March largely differed between sampling years in Cesantes, suggesting an alteration on the meadow status, which could be associated to extensive macroalgae proliferation. The relative abundance of *Crenarchaeota*, *Desulfobacterota*, *Campylobacterota*, *Spirochaetota*, and *Modulibacteria* in *Z. marina* roots was relatively higher in Cesantes than in Bouzas, suggesting a more active role of N_2_ fixation, nitrification and S cycling in Cesantes.

**Conclusions:**

Our results suggest that the seagrass microbiome may respond to environmental conditions and suggest that the temporal monitoring of the prokaryotes associated to roots and leaves may be a valuable tool to assess the seagrass meadow ecological and conservation status.

**Supplementary Information:**

The online version contains supplementary material available at 10.1186/s40793-025-00760-z.

## Background

Seagrasses are marine angiosperms that inhabit sheltered, shallow coastal waters globally playing a crucial role in providing essential ecosystem services [57]. Among others, these services encompass habitat provision and nursery grounds, coastal protection, and the sequestration of atmospheric CO_2_ into marine sediments [7, 17, 23, 40]. Despite their vital ecological function, seagrass ecosystems are increasingly under threat from anthropogenic pressures, including mechanical disturbances, nutrient over-enrichment, and the far-reaching impacts of climate change [60, 80].

Seagrass meadows create complex habitats that support a wide variety of life forms, colonizing both the plant surfaces and the surrounding sediments [19]. This biological complexity extends beyond multicellular organisms, as diverse microbial communities intricately associate with the plants and their environment. These microbes interact with their hosts in various ways, collectively forming holobionts or metaorganisms [12, 73, 75]. Such interactions are thought to be fundamental to the functioning and resilience of these ecosystems, underscoring the interconnectedness and dependencies that sustain seagrass habitats, enabling them to support biodiversity and provide essential ecosystem services, such as reducing pathogens and toxic dinoflagellates in the surrounding water [16, 36, 37, 74]. Recently, several culture-independent surveys of seagrass microbiomes have been published, providing valuable initial reference points for further work [14, 25, 38, 50, 52, 72, 77]. While these studies reveal similar overarching findings, such as the potential existence of core microbiomes, clear differences in microbiome composition between the referred studies highlight the need for further investigation.

*Zostera marina* is the most widespread seagrass in temperate coastal zones and estuaries in the North Atlantic [68]. It plays a vital ecological role in these ecosystems, where seasonal changes strongly influence its growth patterns and physiological responses. In temperate regions, *Z. marina* experiences marked seasonal fluctuations, with rapid growth occurring in spring and summer when water temperatures and light availability increase, followed by a decline in biomass during the colder months [5]. These seasonal dynamics are critical for nutrient cycling and habitat structure [59].

The *Z. marina* microbiome plays a crucial role in maintaining the health, resilience, and ecological function of this seagrass species, particularly in temperate ecosystems [22]. Research on *Z. marina* and other temperate seagrass species has shown that the microbial communities associated with both the leaves and roots contribute significantly to nutrient cycling, plant defense, and overall stress tolerance [14, 73]. Leaf microbiomes, exposed to the surrounding water column, tend to be more variable than root microbiomes and to some extent they appear to be related to seawater microbiomes [22, 65]. In contrast, root microbiomes, embedded in more stable and heterogeneous sediment environments, are more diverse and less affected by these fluctuations but still undergo shifts due to changes in sediment chemistry and organic matter input [22, 78]. Despite these observations, the temporal succession of these microbial communities across different growth stages of the meadow remains poorly understood [42]. How these microbiomes adapt to dynamic environmental factors, such as temperature shifts and nutrient availability during different seasons, and how anthropogenic impacts further shape these interactions, remain poorly understood, leaving critical gaps in our understanding of the precise roles microbial communities play in plant health and overall seagrass ecosystem dynamics.

Founded on these considerations, we hypothesize that the microbial communities associated with *Z. marina* leaves and roots will exhibit distinct diversity and temporal patterns associated to distinct environmental conditions and anthropogenic pressures.

To test this hypothesis, we explored the temporal variation of leaf and root microbiome composition of populations of *Z. marina* located in two nearby coastal temperate zones in the Ría de Vigo (NW Spain), which potentially differ in anthropization levels: Bouzas beach, located in a highly urbanized area, and Cesantes beach, located in a rural area. The study seeks to achieve the following objectives: (i) describe the microbial diversity and taxonomic composition in *Z. marina* leaves and roots; (ii) compare microbial community composition between two sites characterized by different anthropic influence (Bouzas and Cesantes), and (iii) analyze the diversity and temporal patterns of leaf and root microbiomes and how these patterns are related with seagrass traits and meadow environmental settings.

## Methods

### Sample collection

**Fig. 1:**
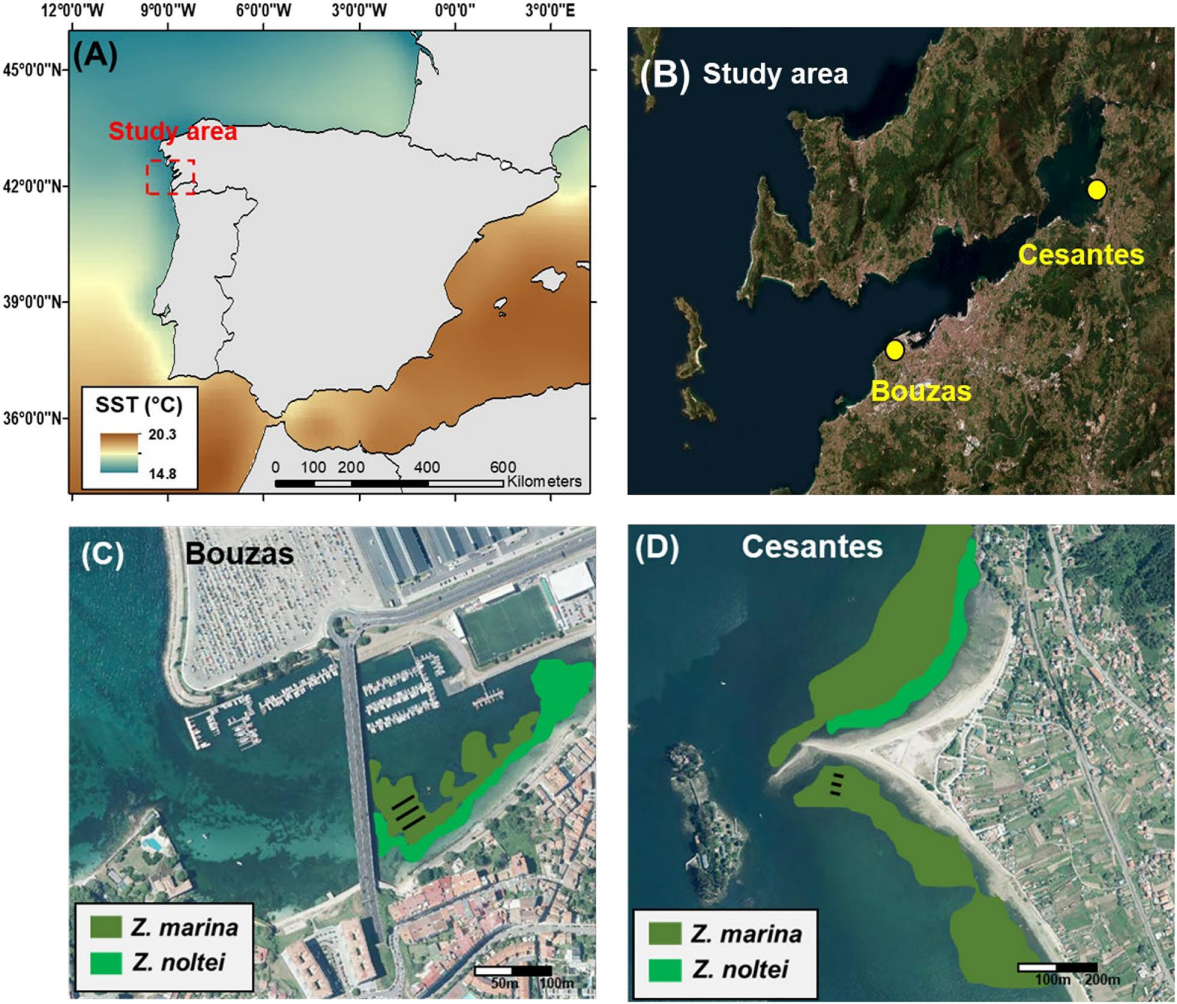
Maps showing the study area and the sampling locations in the Ría de Vigo (NW Spain) (**A**, **B**). Maps depicting the approximate distribution of Zostera marina and Zostera noltei meadows in the sampling areas in May 2022, with black lines representing the three sampling transects (**C**, **D**)

Plants were collected from Bouzas (Latitude: 42.225, Longitude: − 8.757) and Cesantes (Latitude: 42.308, Longitude: − 8.625) in March 2021, April 2021, July 2021, and March 2022, during low spring tides. Both sites are situated in NW Spain, within the Ría de Vigo, a temperate estuarine system influenced by strong seasonal upwelling events (Fig. 1). The Ría is subjected to significant seasonal variability, with water temperatures ranging from approximately 11 °C in winter to 21 °C in summer [6].

In the Bouzas site, the upper intertidal zone is dominated by *Zostera noltei*, while the lower intertidal and subtidal zones are primarily colonized by *Zostera marina*. In the sampling site in Cesantes, *Zostera marina* occupies both the intertidal and subtidal zones. Bouzas, located in the central part of the Ría near the city of Vigo and close to the industrial and commercial Port of Vigo, experiences a high degree of anthropogenic pressure, influenced by urban runoff and extensive human activity on a heavily impacted beach. Conversely, Cesantes, positioned further inland within the Ría, is subject to lower anthropogenic influence and faces less urbanization and industrialization. A detailed spatial distribution of land cover classes in the surroundings (1,000 m radius) of the two sampling sites supports the higher level of anthrophization in Bouzas compared to Cesantes (Figure S1). As a consequence of this anthropogenic influence, the sediments close to the marina of Bouzas, near the *Zostera marina* meadow, showed the highest level of copper concentration reported in a study conducted in the Ría de Vigo [24]. Both locations present a seasonal variation in seawater temperature, and temporal variations in salinity associated with freshwater inputs, particularly in Cesantes (Figure S2).

At each sampling event, three 30-m transects were established within the meadows, representing the subtidal, upper intertidal, and middle intertidal zones (Fig. 1C-D). Along each transect, three sampling areas were established, spaced ca. 15 m apart from each other. In each of the nine areas, between 5 and 10 plants were randomly collected. The distance between the transects was also set at 15 m to ensure sufficient spatial coverage across the different zones. Following collection, the plants were immediately placed in seawater-filled containers and transported to the laboratory under dark conditions to maintain their physiological and morphological integrity, ensuring accurate data for further analysis.

### Plant and meadow characteristics

Morphometric descriptors were evaluated by randomly selecting 10–15 healthy shoots along each transect during each sampling, with a spacing of 2–3 m between shoots. Measurements included number of leaves per shoot (no. leaves shoot^−1^), leaf area (cm^2^ shoot^−1^), and rhizome length (cm). Alongside, density (shoots m^−2^) was calculated from 30 cm^2^ sampling units and later normalized to m^2^.

Growth was estimated from leaf elongation (cm m^−2^ d^−1^) using the 'punching' method described by Short and Duarte [67]. Two small holes were made in each leaf with a hypodermic needle, spaced 2–3 mm apart above the plant's basal meristem. A total of 10–15 shoots were randomly selected and marked in each meadow. After a period of 28–31 days, the marked shoots were harvested and taken to the laboratory. Leaf elongation was calculated by measuring the distance between the two punched holes and the meristem for each growing leaf. Relative growth (d^−1^) was estimated as leaf area production (cm^2^ shoot^−1^ d^−1^) divided by leaf area (cm^2^ shoot^−1^). Additionally, the percentage of necrosis was determined for the second and third youngest leaves on each plant by estimating the proportion of damaged tissue compared to the total leaf area. This was done by dividing the leaf into sections and visually assessing the percentage of the leaf surface affected by necrosis.

The biomass of macroalgae in the meadows was estimated from samples collected in one area per transect using a 0.30 m^2^ quadrat. All biomass within the quadrat, including seagrasses and macroalgae, was harvested. In the laboratory, the samples were carefully processed by separating macroalgae from seagrasses and dead material. To determine the dry weight of the macroalgae biomass, the samples were dried at 60 °C for seven days and subsequently weighed. The resulting biomass data were normalized to a square meter.

### Photosynthetic efficiency

The photosynthetic efficiency of leaves maintained in dark conditions was measured 20–40 min after collection using a portable Handy Pea (Hansatech) fluorometer (March 2021, April 2021) or a Junior PAM (Walz) fluorometer (July 2021, March 2022) using appropriate leaf clips. In July we conducted duplicate measurements to intercalibrate both instruments rendering a significant (ANOVA, *p* < 0.001) regression (slope = 1.1 ± 0.06, r^2^ = 0.88). Both instruments allow the full range of saturation pulse analysis of photosystem II. The maximum photochemical yield of photosystem II (PSII), Fv/Fm, was measured in the second youngest leave, and three shoots were used per area. The mean from the three replicates was calculated for each of the nine areas in each meadow and sampling date.

### Carbon and nitrogen content in leaves and roots

The nitrogen (N) and carbon (C) concentrations in leaves and rhizomes (%) were measured from approximately 7.0 mg of freeze-dried, finely ground, and homogenized material per sample in triplicate. Triplicate samples were collected from two randomly selected areas (one in March 2021) in each sampling period. The N and C content in the leaf and rhizome samples was analyzed through high-temperature catalytic oxidation at 900 °C using a PerkinElmer 2400 elemental analyser and expressed as percentage of dry weight. To determine the dry weight, the biomass was dried at 60 °C for 48 h in an oven [67]. The mean variation coefficient for C content was 3.8% and 8.1% for leaves and rhizomes, respectively. The mean variation coefficient for N content was 8.9% and 17.5% for leaves and rhizomes, respectively.

### Sediment properties and porewater nutrient analysis

At each sampling area, five undisturbed sediment mini-cores (2.5 cm diameter) were collected by hand. Cores collected from each area were sliced on site (0–1, 1–3, 3–6 and 6–10 cm depth) and equivalent depths were gently mixed inside zip bags. Sediment subsamples were collected and stored frozen to determine sediment properties. The remaining sediment was then used to extract porewater samples using Rhizon Soil Moisture Samplers (Rizhosphere, The Netherlands) following the cautions presented in Ibánhez and Rocha [34]. Nitrate, nitrite, phosphate and silicate concentrations in the porewater were measured following standard colorimetric methods on an Alliance Futura segmented flow autoanalyzer [27]. For ammonium, the fluorometric method of Kérouel and Aminot [41] was used. The concentration of nutrients was then integrated for the sampled sediment column (10 cm).

Sediment organic matter content was determined in the laboratory using the frozen sediment subsamples. Total organic matter content was determined in one area per transect using the loss on ignition determination [15].

### Microbiome sampling

Only one area per transect was sampled in March 2021, while the nine areas were sampled in the other three sampling events. Plants for microbiome analyses were carefully washed with 0.2 μm filtered seawater to remove microbes not attached to the plant. Thereafter leaves and rhizomes/roots were separately stored in sterile plastic bags and flash frozen in liquid N_2_ for 20 min. Finally, samples were stored at -80 ºC until further processing.

### DNA extraction and sequence analysis

Only one shoot per section was processed for microbiome analysis. 0.25 g of the apical part of the second youngest leave or roots were cut under sterile conditions and DNA was extracted with DNeasy® PowerWater® Kit (Qiagen, Germany) following the manufacturer´s protocol. The DNA concentration was quantified using Qubit 2.0. fluorometer and the Qubit dsDNA HS assay kit (Thermo Fischer Scientific, MA, USA). Mean DNA concentration in Bouzas and Cesantes leaves was, respectively, 3.7 ± 0.6 and 2.1 ± 0.3 ng/µl, and DNA concentration in Bouzas and Cesantes roots was, respectively, 10.6 ± 0.9 and 10.3 ± 1.0 ng/µl. Prokaryote community composition was assessed by sequencing the V4-V5 hypervariable region of the 16S rRNA gene using Illumina Miseq platform and a minimum of 2 × 250 bp paired-end reads at the Fasteris Laboratory (Geneva, Switzerland), using the following primers: 515F-Y (5’-GTGYCAGCMGCCGCGGTAA-3’) and 926-R (5’-CCGYCAATTYMTTTRAGTTT-3’) [61].

DNA sequence reads were processed according to the MiSeq amplicon analysis workflow described by Logares [49]. Initially, raw reads were corrected using BayesHammer [56, 66]. Following this, paired-end reads were merged with PEAR [85] and then subjected to quality control filtering and length check using USEARCH [20]. Next, sequences were aligned in the correct 5’-3’ direction and checked for rDNA signatures using Hidden Markov Model (HMM), following the miTags protocol [48]. Subsequently, dereplication was performed using VSEARCH v2.14.1 [64]. Afterward, the reads were sorted by abundance, singletons were discarded, and clustering was performed whith UPARSE v1.5 [21]. Then, chimeric sequences were identified using the SILVA_138_SSURef_Nr99 reference database [62] and removed. Thus, operational taxonomic units (OTUs) were obtained by clustering reads with 99% similarity. Thereafter, reads were mapped back to the OTUs to estimate abundance, and an OTU table was generated. Finally, OTUs were taxonomically classified using BLAST [3] against the SILVA database. OTUs with < 200 bp alignment, < 60% coverage, < 90% similarity, and > 0.00001 e-values were removed, as described in [26, 39]. Taxonomic assignment and the OTU table were merged using the R software (R Foundation for Statistical Computing, 2023). All OTUs assigned to chloroplasts, mitochondria, or eukaryotes were removed. The Vegan Package (Oksanen, 2022) in R was used for subsampling the OTU table to the lowest number of reads, 1,945. These sequences are publicly available at the NCBI GenBank database under accession number PRJNA1210619.

### Statistical analyses

A two factor ANOVA test was used to test for differences in microbial diversity and environmental variables between locations and sampling periods for leaves and roots separately. Bonferroni test was used for post-hoc comparisons. Data were log transformed when necessary to fit a normal distribution. Kruskal–Wallis (K-W) test was applied when normality was not attained (e.g. nutrient concentrations). T-test was used for comparisons of macroalgae biomass, growth, C and N content, necrosis and sediment organic matter between the two locations.

The OTU table was subsampled using the rrarefy function in the Vegan R package [58], and used for calculating richness (S), evenness (Pielou’s J), and Shannon diversity (H). The Vegan package [58] was used for calculating relative abundance of different taxa across each sample. The web-based tool InteractiVenn was used for the analysis of sets through Venn diagrams [30]. The principal coordinate analysis (PCoA) using Bray–Curtis similarities was applied to visualize sample ordination based on the taxonomic composition of the microbial community. The PERMANOVA test was used to test for significant differences among sample groups. SIMPER test was used to determine which of the top 100 OTU (which contributed 40% to total read abundance) were responsible for the differences in composition between leaves and roots and between Bouzas and Cesantes. Relative abundance heatmaps were constructed using the R script ComplexHeatmap [28]. Correlation heatmaps and associated clustering were constructed using Morpheus analysis software (https://software.broadinstitute.org/morpheus). Pearson correlation was used to assess the relationship between standardized diversity, morpho-/physiological and biogeochemical variables (sediment nutrient concentration). In this case the clr abundance of major taxonomic groups was used to avoid spurious correlations. The average Euclidean distance was used for clustering of samples and variables in the correlation heatmaps.

## Results

### Sediment porewater nutrient concentrations and sediment organic matter

**Fig. 2:**
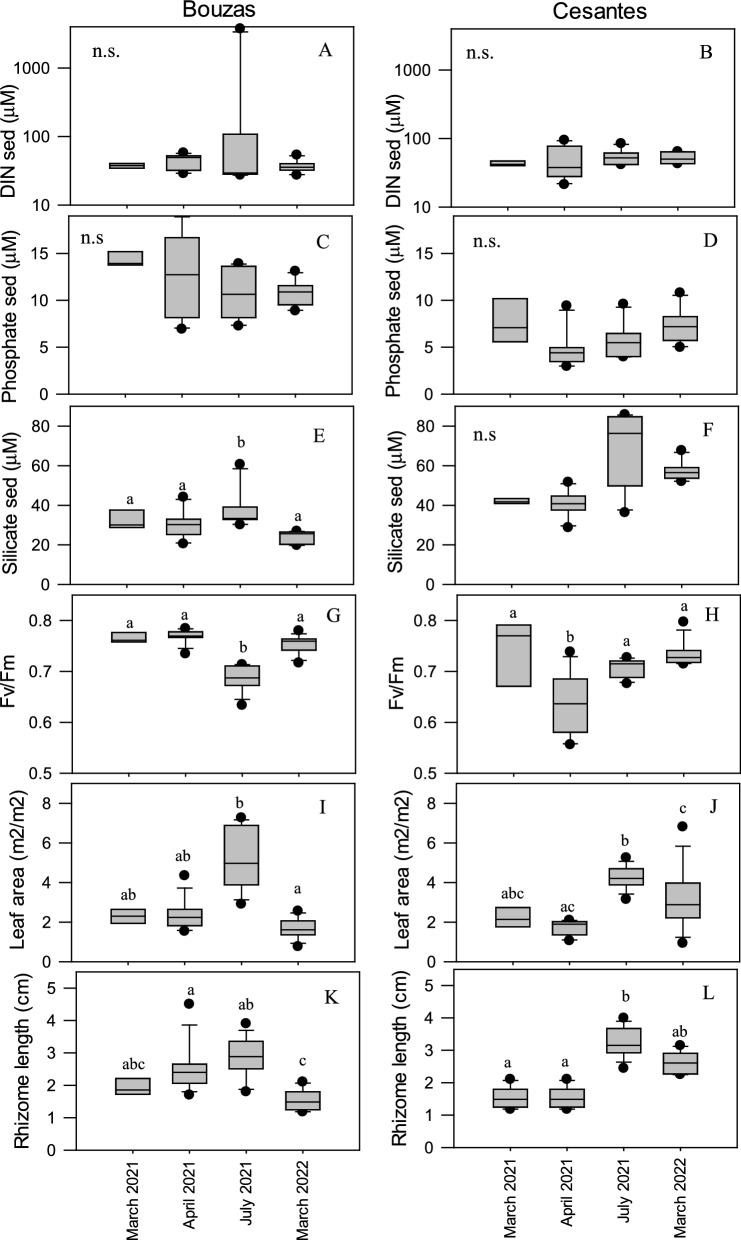
Box plots showing the concentration of nutrients in sediment porewater fluids (DIN: Dissolved Inorganic Nitrogen; Phosphate and Silicate), as well as the photosynthetic efficiency (Fv/Fm), the leaf area, and the rhizome length of plants in Bouzas (**A**, **C**, **E,** **G**, **I**, **K**) and Cesantes (**B**, **D**, **F**, **H**, **J**, **L**) during the four sampling periods. Lines within the plots represent the mean value. The bottom and top boundaries of each boxplot indicate the 25th and 75th percentiles, respectively. The whiskers are the 5th and 95th percentiles, respectively. Letters in the boxes indicate significantly equal mean values within each sampling site according to the post hoc tests (*p*-value < 0.05). n.s: not significant differences among sampling periods

Depth-integrated sediment porewater dissolved inorganic nitrogen (DIN) concentration, estimated as the sum of nitrate, nitrite an ammonium, ranged from 21 μM in Cesantes beach in April 2021 to 3,729 μM in Bouzas beach in July 2021 (Fig. 2A-B).

**Fig. 3:**
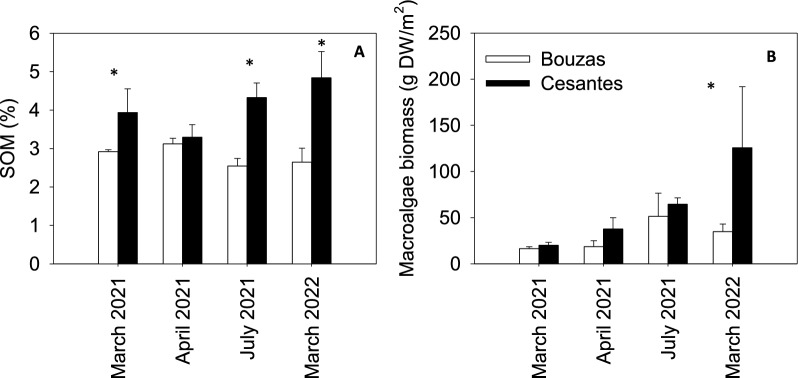
Sediment organic matter (SOM) (**A**) and macroalgae biomass (**B**) in Bouzas and Cesantes meadows during the four sampling periods. Error bars represent standard errors. Asterisks indicate significant differences between locations

On average, 93% of the DIN was in the form of ammonia (data not shown). Depth-integrated DIN concentration did not differ between both locations (K-W, *p* = 0.320). By contrast, depth-integrated phosphate concentration was significantly higher in Bouzas (11.9 ± 0.7 μM) than in Cesantes (6.5 ± 0.5 μM) (K-W, *p* < 0.001), while silicate was significantly higher in Cesantes (52 ± 4 μM) than in Bouzas (31 ± 2 μM) (K-W, *p* < 0.001) (Fig. 2C-F). Nutrient concentrations did not significantly differ among sampling periods in Bouzas (K-W, *p* > 0.09) while silicate concentration was significantly higher in July 2021 than in the other sampling periods in Cesantes (K-W, *p* = 0.026). The content of organic matter in the sediment was higher in Cesantes than in Bouzas (t-test, *p* < 0.05), except in April 2021 (Fig. 3A).

### Plant and meadow characteristics

The density of shoots varied from 172 to 340 shoots m^−2^, and there were no significant differences between meadows (Table S1). The maximum photosynthetic efficiency of the PSII (Fv/Fm) of the plants was significantly higher in Bouzas (0.74 ± 0.1) than in Cesantes (0.70 ± 0.01) (ANOVA *p* < 0.001) (Fig. 2G-H) and showed minimum values in July 2021 in the Bouzas meadow and in April 2021 in the Cesantes population. There were no significant differences in leaf area per m^2^ of meadow (Fig. 2I-J) nor in growth (Table S1) between locations, while rhizome length (Fig. 2I-J) was significantly lower in Bouzas than in Cesantes (ANOVA, *p* = 0.034). Seasonal growth patterns differed between locations (Fig. 2K-L). In Bouzas, both leaf area and rhizome length were maximum in July 2021 and minimum in March 2021 and March 2022 (i.e., at the beginning of the growing season). By contrast, rhizome length was higher in March 2022 than in March 2021 in Cesantes.

Mean necrosis in leaves did not differ between locations and was overall higher in March 2021 than in the other sampling periods (Table S1). The macroalgae biomass associated to the meadow was similar at both locations with the exception of March 2022, when the biomass was significantly higher in Cesantes (Fig. 3B). C and N content in leaves was similar between both locations, rendering a mean C: N of 22 ± 2 in Bouzas and 21 ± 2 in Cesantes (Table S2). By contrast, N content in rhizomes was significantly higher in Cesantes than in Bouzas (t-test, p < 0.001), resulting in a mean C: N of 60 ± 7 in Bouzas and 32 ± 3 in Cesantes (Table S2).

### Microbiome diversity in leaves and roots of *Zostera marina*

**Fig. 4:**
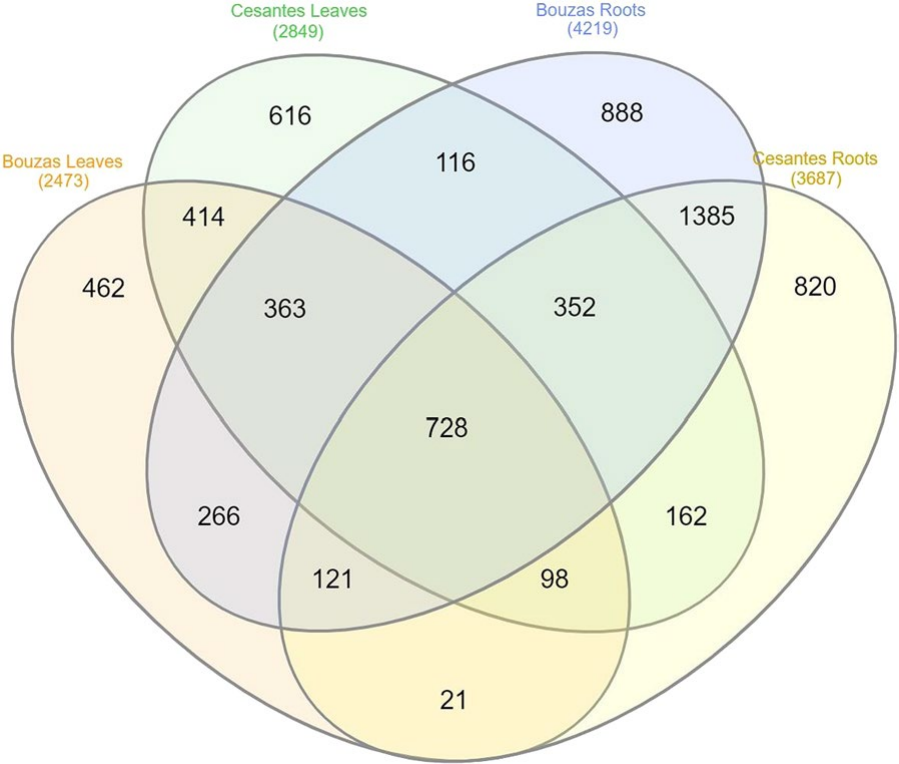
Venn diagram showing exclusive and common prokaryote taxa among Bouzas leaves, Cesantes leaves, Bouzas roots and Cesantes roots

The total number of different OTUs detected after subsampling was 6,812, of which, 3093 (45%) were exclusively present in roots and 1,492 (22%) were only present in leaves (Fig, 4). Most OTUs (53%) were common among locations, while 24% and 23% were uniquely present in Bouzas or Cesantes, respectively (Fig. 4). Only 728 OTUs were present in both locations and plant parts. The number of different OTUs was higher in roots (5,321) than in leaves (3,719) (data not shown), of which, 49%, for roots, and 43%, for leaves, were common to both locations (Fig. 4).

**Fig. 5:**
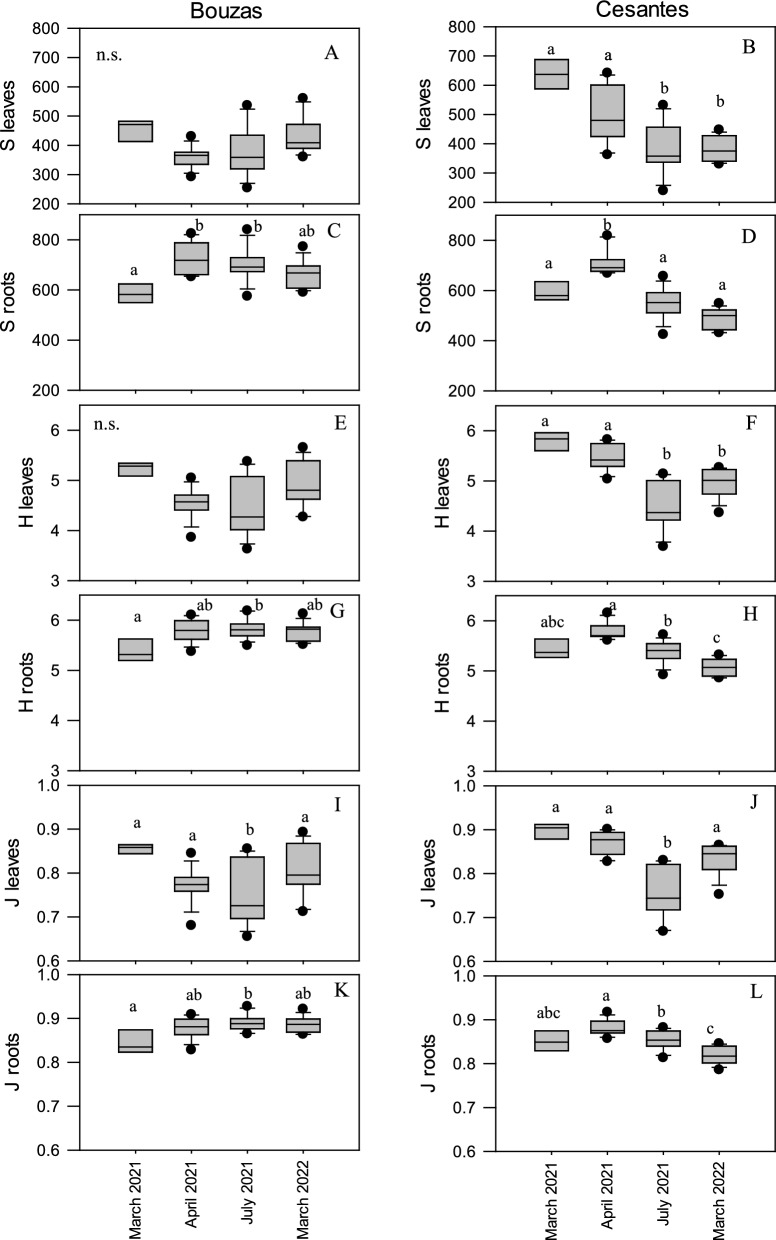
Box plots showing the richness (S), Shannon diversity index (H) and Pielou’s equitability index (J) in Bouzas (**A**, **C**, **E**, **G**, **I**, **K**) and Cesantes (**B**, **D**, **F**, **H**, **J**, **L**) during the four sampling periods. Lines within the plots represent the mean value. The bottom and top boundaries of each boxplot indicate the 25th and 75th percentiles, respectively. The whiskers are the 5th and 95th percentiles, respectively. Letters in the boxes indicate significantly equal mean values within each sampling site according to the post hoc tests (*p*-value > 0.05). n.s: not significant differences among sampling periods

OTU richness (S) in leaves ranged from 239, in July 2021 in Cesantes, to 705, in March 2021 in Cesantes, and mean OTU richness was higher in Cesantes than in Bouzas (ANOVA, *p* = 0.003) (Fig. 5A-B). S in roots ranged from 424, in July 2021 in Cesantes, to 840, in July 2021 in Bouzas, and mean OTU richness was significantly higher in Bouzas (688 ± 13) than in Cesantes (585 ± 19) (ANOVA, *p* < 0.001) (Fig. 5C-D). In Bouzas, mean S was minimum in leaves and maximum in roots in April 2021. In Cesantes a significant downward trend in S was observed both for leaves and roots along the study period. Shannon diversity (H) and Pielou’s index (J) followed very similar patterns to those observed in S (Fig. 5E-L). Overall, S, H and J were more variable in leaves than in roots.

### Microbiome composition in leaves and roots of *Zostera marina*

**Fig. 6:**
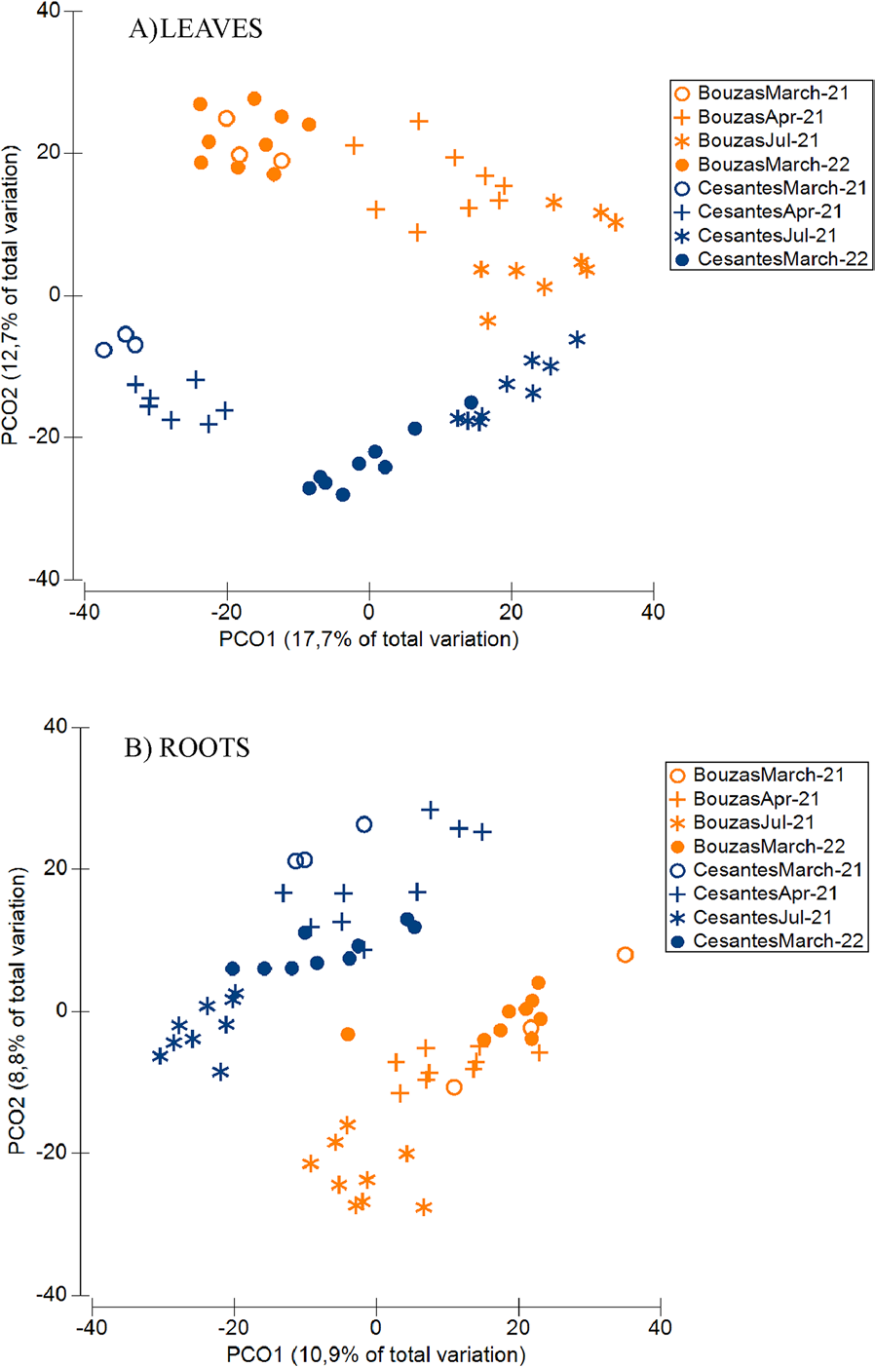
Principal coordinate analysis showing ordination of samples according to prokaryote community composition in leaves (**A**) and roots (**B**)

There were significant differences in the microbial community composition between roots and leaves (PERMANOVA, *p* = 0.001), between sites (PERMANOVA, *p* = 0.001), and between sampling dates (PERMANOVA, *p* = 0.001) (Fig. S3-S4, Fig. 6). The interaction between these three factors was also significant (PERMANOVA, *p* = 0.001). By contrast, there were no significant differences in the microbial community composition among intertidal levels (PERMANOVA, *p* = 0.462 for roots, and *p* = 0.835 for leaves). The microbiome of leaves was significantly different between locations (PERMANOVA, *p* = 0.001) (Fig. 6A), although the mean distance between sites was minimum in July 2021. In Cesantes, community composition was significantly different among all sampling dates (PERMANOVA, *p* < 0.008), while in Bouzas, there were no significant differences in the microbiome of March 2021 and March 2022 (PERMANOVA, *p* = 0.056). The microbiome of roots was significantly different between sites (PERMANOVA, *p* = 0.001), and the seasonal patterns were less marked than in leaves (Fig. 6B). In Cesantes, root microbiome did not significantly differ between March 2021 and April 2021 (PERMANOVA, *p* = 0.137). In Bouzas, only the microbiome of July 2021 significantly differed from that of the other sampling dates (PERMANOVA, *p* < 0.025).

**Fig. 7:**
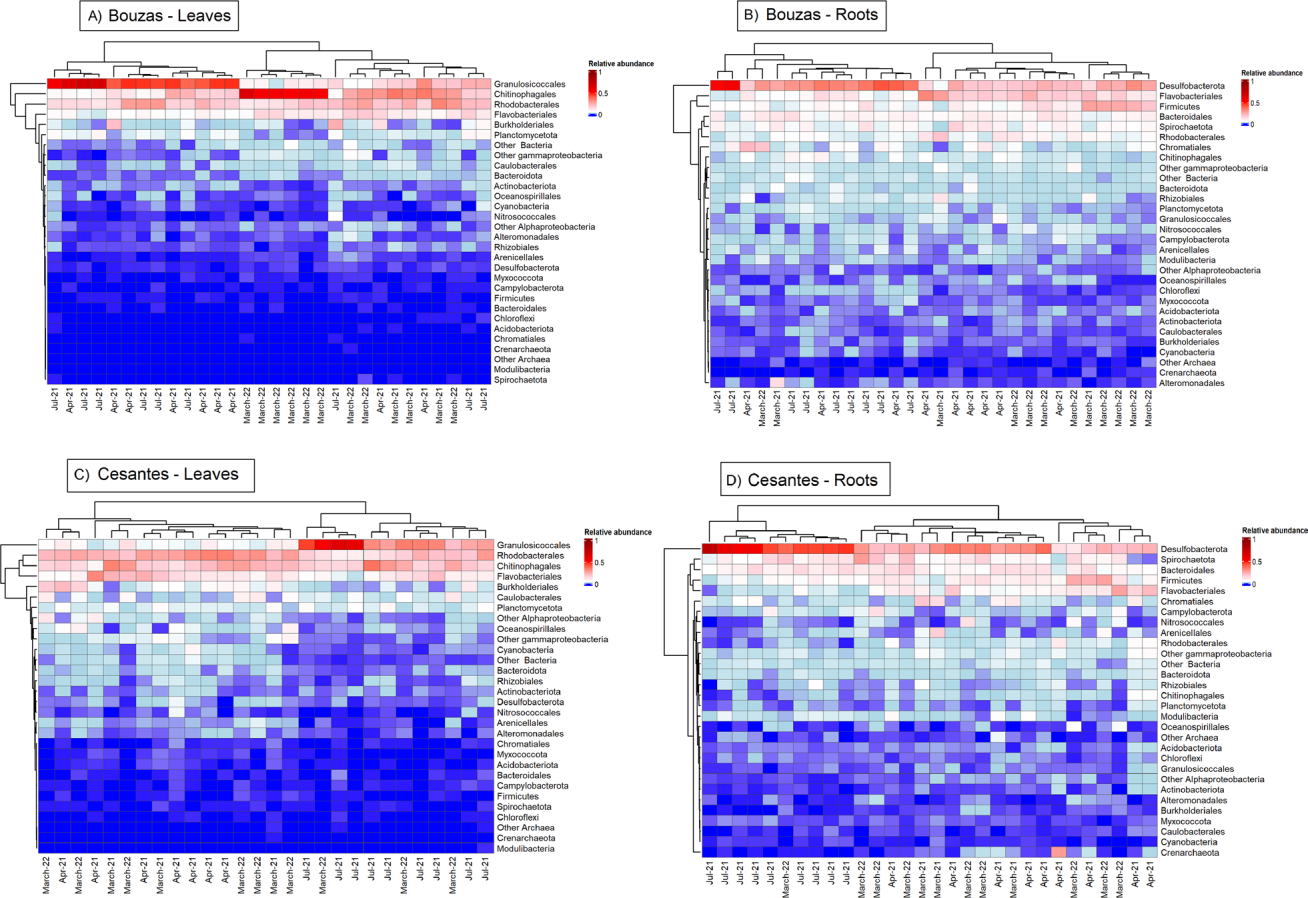
Heatmap of the relative abundance of the main prokaryote groups (including phyla to orders) in Bouzas leaves (**A**), Bouzas roots (**B**), Cesantes leaves (**C**), and Cesantes roots (**D**) in each sample during the four sampling periods. The dendrograms in the margins of each heatmap represent hierarchical clustering based on Euclidean distance complete linkage

*Granulosicoccales* (class *Gammaproteobacteria*), *Rhodobacterales* (class *Alphaproteobacteria*), *Chitinophagale*s (phylum *Bacteroidota*) and, to a lesser extent, *Flavobacteriales* (phylum *Bacteroidot*a), dominated the microbiome of leaves at both locations (Fig. 7A, C). *Caulobacterales* (class *Alphaproteobacteria*), *Planctomycetota*, and *Burkholderiales* (class *Betaproteobacteria*) were also relatively abundant in leaves. *Granulosicoccales* tended to be more abundant in April 2021 and July 2021 in Bouzas, and in July 2021 in Cesantes. *Granulosicoccales* were relatively more abundant in Bouzas than in Cesantes, while many other less dominant groups, such as *Chloroflexi*, *Spirochaetota*, *Acidobacteriota*, *Desulfobacterota*, *Cyanobacteria*, *Chromatiales* (class *Gammaproteobacteria*), *Arenicellales* (class *Gammaproteobacteria*), *Oceanospirillales* (class *Gammaproteobacteria*) and *Caulobacterales* (class *Alphaproteobacteria*), were relatively more abundant in Cesantes than in Bouzas.

*Desulfobacterota*, *Flavobacteriales* (phylum *Bacteroidota*), *Bacteroidale*s (phylum *Bacteroidota*), *Firmicutes*, and *Spirochaetota* were the dominant bacterial taxa on roots (Fig. 7B, D). Several *Alphaproteobacteri*a orders (*Rhodobacterales*, *Rhizobiales*, *Caulobacterales*), two *Gammaproteobacteria* orders (*Alteromonadales*, *Granulosicoccales*), *Planctomycetota*, *Cyanobacteria*, *Flavobacteriales* (*Bacteroidota*) and *Chitinophagales* (*Bacteroidota*) were more abundant in Bouzas than in Cesantes. On the other side, *Crenarchaeota*, other *Archaea*, *Campylobacterota*, *Desulfobacterota*, and *Modulibacteria*, were more abundant in Cesantes than in Bouzas.

According to the SIMPER analysis, 22 of the top 100 most abundant OTUs explained 50% of the differences between the microbiome of leaves and roots and 53% of the differences between Bouzas and Cesantes (Fig. S5). *Granulosicoccus*_1 (class *Gammaproteobacteria*), and *Saprospiraceae*_1 (phylum *Bacteroidota*), more abundant in leaves and in Bouzas, explained, respectively, 6.35% and 4.02% of the differences between plant parts and 7% and 4% of the differences between locations, while *Spirochaeta*2_1 (phylum *Spirochaetota*), and *Desulforhopalus*_1 (phylum *Desulfobacterota*), dominant in roots and in Cesantes, explained, respectively 5.35% and 4.79% of the differences between plant parts and 5.87% and 5.83% of the differences between locations. In addition, two *Rubidimonas* OTUs, were exclusively found in Bouzas, contributing to 3% of the top 100 abundance in their leaves.

### Links between microbiome composition and diversity indices, plant characteristics and sediment porewater nutrients

**Fig. 8:**
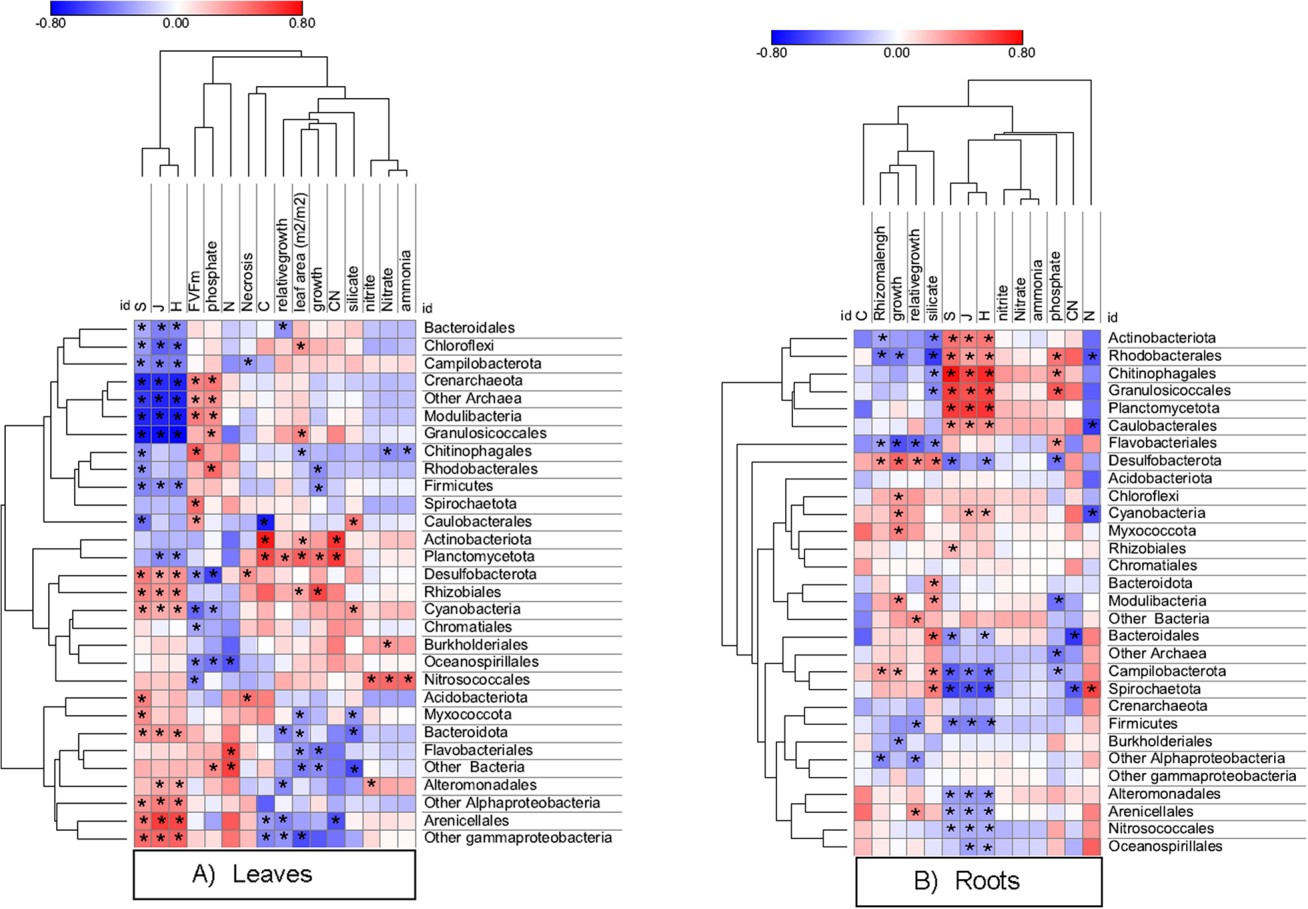
Pearson correlation heatmap of the main microbial taxa in leaves with sediment nutrient concentrations (nitrite, nitrate, ammonia, silicate and phosphate), leaf diversity indices (S, J, H), plant photosynthetic efficiency (Fv/Fm), necrosis, growth, relative growth, and C, N and C:N content of leaves (**A**). Pearson correlation heatmap of the main microbial taxa in roots with sediment nutrient concentrations (nitrite, nitrate, ammonia, silicate and phosphate), root diversity indices (S, J, H), rhizome length, growth, relative growth, and C, N and C:N content of rhizomes (**B**). The dendrograms represent clustering of taxa and variables based on average Euclidean distance. Asterisks indicate statistically significant Pearson correlations (*p*-value < 0.05)

Pearson correlation revealed a cluster of prokaryotes negatively related with microbiome diversity of leaves and positively related to the photosynthetic efficiency of the plant (Fv/Fm) (Fig. 8A), including *Bacteroidales* (phylum *Bacteroidota*), *Chloroflexi*, *Campylobacterota*, *Crenarchaeota*, *Modulibacteria*, *Granulosicoccale*s (class *Gammaproteobacteria*), *Chitinophagales* (phylum *Bacteroidota*), *Rhodobacterales* (class *Alphaproteobacteria*), *Firmicutes*, *Spirochaetota* and *Caulobacterales* (class *Alphaproteobacteria*). *Actinobacteriota* and *Planctomycetota* clr abundance was positively related with leaf area, and both leaf C content and leaf C: N. A second important cluster of prokaryotes included *Desulfobacterota*, *Rhizobiales* (class *Alphaproteobacteria*), *Cyanobacteria*, *Chromatiales* (class *Gammaproteobacteria*), *Burkholderiales* (class *Betaproteobacteria*), *Oceanospirillales* (class *Gammaproteobacteria*) and *Nitrosococcales* (class *Gammaproteobacteria*), which mostly related positively with diversity and negatively with Fv/Fm. *Nitrosococcales* strongly and positively related with nitrate, nitrite and ammonia in porewaters.

In the case of the root microbiome (Fig. 8B) a cluster including *Actinobacteria*, *Rhodobacterales* (class *Alphaproteobacteria*), *Chitinophagales* (phylum *Bacteroidota*), *Granulosicoccales* (class *Gammaproteobacteria*), *Planctomycetales* (phylum *Planctomycetota*) and *Caulobacterales* (class *Alphaproteobacteria*) appeared strongly and positively related to diversity indices and negatively to silicate concentration in porewaters. By contrast, *Campylobacterota*, *Spirochaetota*, *Alteromonadales* (class *Gammaproteobacteria*), *Arenicellales* (class *Gammaproteobacteria*) and *Nitrosococcales* (class *Gammaproteobacteria*) were negatively related with diversity indices. Notably, several root microbial phyla were positively related with plant growth, including *Desulfobacterota*, *Chloroflexi*, *Cyanobacteria*, *Myxococcota*, *Campylobacterota*, and *Modulibacteria*.

## Discussion

According to our expectations, and as previously reported in *Z. marina* and other seagrasses, microbiome diversity was higher in roots than in leaves, and prokaryote composition was more variable in leaves than in roots (e.g., [22, 63, 65, 78]). The higher diversity and the higher proportion of exclusive taxa in roots than in leaves is likely related to a wider variety of micro-niches around the roots, including redox gradients, allowing the recruitment of metabolically diverse microbial taxa [18, 75].

Sulfate-reducing, nitrogen-fixing *Desulfobacterota* and *Spirochaetota*, including mostly anaerobic bacteria, were preferentially present in the root microbiome. Both taxa had been previously reported as indicator microbes of rhizome and root tissues in seagrasses, reflecting the predominately anoxic conditions of seagrass sediments [2, 22, 32, 50, 63, 75]. *Desulfobacterota* and *Spirochaetes*-like bacteria, more abundant in the roots of plants from Cesantes than in those from Bouzas, have been identified as potentially relevant players in mercury methylation in low-oxygen sediments [9], suggesting a higher potential for production of this neurotoxic compound in the less anthropized meadow. Even though there is no published information about mercury or methylmercury in the studied sediment porewaters, the total amount of mercury and methylmercury in the sediment has been seen to be higher in Bouzas than in Cesantes [8]. Notwithstanding, a recent survey (2023–2024) analysing porewaters of eight different beaches along the Ría de Vigo found the highest total (> 20 pM) and methylmercury concentrations at the Cesantes beach (Andrea G. Bravo, personal communication).

The most representative group of leaf microbiome in the studied *Z. marina* meadows was the order *Granulosicoccales*. Members of this order had been previously identified as core taxa in *Zostera* leaves [13, 32, 43, 65] and also as dominant microbes on the leaves of seaweeds [45, 81], nonetheless, its role within the holobiont remains unclear. The detailed description of the genome of kelp-associated *Granulosicoccus* by Weigel et al. [81] revealed that they are extremely versatile motile bacteria, with potential for photoheterotrophy, nitrogen- and sulfur-cycling, and cobalamin (B12) synthesis. A recent study based on the assemblage of a large number of prokaryotic genomes from macroalgae microbiomes found that *Granulosicoccales* contained the largest number of gene clusters potentially associated to degradative pathways of anthropogenic organohalogens [44], which would be coherent with the higher abundance of this order in Bouzas compared to Cesantes. The exclusive presence of two relatively abundant *Rubidimonas* OTUs in Bouzas suggest a distinct status of the two meadows, which in part could be related to the differential level of anthropogenic pressures. Members of the *Rubidimonas* genus appear to be aerobic heterotrophs that can metabolize complex starch molecules [51] and have been associated to seagrass wasting disease [33]. Methylotrophic bacteria, capable of metabolizing the methanol produced by the plant [54], are also typically found in the core microbiome of seagrass leaves [1, 13]. The *Burkholderiales* detected in this study, including mostly methylotrophic bacteria, were more abundant in leaves than in roots in both locations. *Caulobacterales*, which have been found to play an important role as decomposers of lignin, cellulose and hemicellulose [83], and *Rhodobacterales*, *Alteromonadales* or *Actinobacteriota*, reported to include algicidal bacteria [36], were also more represented in leaves than in roots at both locations.

The relatively high proportion of taxa shared between locations (53%) provides support to the previously reported specificity of the seagrass microbiome [25, 65, 75]. Conversely, the differences between the microbiome of these two nearby locations also highlight the influence of the environment on its composition. For example, Sanders-Smith et al. [65] observed that the microbiome was more similar between locations in new leaves, suggesting that the microbiome in the initial phases of leave colonization must be more host-specific. Then, in older leaves, the differences in the microbiome between locations are higher and probably more linked to the surrounding environment. In our case, we focused on the second youngest leave, which might explain the relatively high similarity between the two sampled locations. Specifically, in our study locations, *Zostera marina* plants typically produce one leaf every 12–15 days in winter and every 5–7 days in summer (unpublished data). The hypothesis of plants being more selective during the initial phases of leaf colonization would also explain that the highest similarity in leaf microbiome between locations as well as the lowest diversity occurs in summer, when the growth is also highest, which imply less time for leaf colonization. Sanders-Smith et al. [65] also found lower diversity in new than in older leaves.

Interestingly, more microbial taxa from roots, such as *Desulfobacterota*, *Chloroflex*i, or *Campylobacterota*, appeared to be positively correlated with plant growth than those from the leaves, suggesting the potential of root microbiota to improve the overall performance of seagrasses, possibly through nitrogen fixation or sulfide detoxification [53, 73, 75]. Wang et al. [78] also found a clear link between the root microbiome and the growth of *Z. marin*a in transplant experiments, with lower growth rates associated to the removal of the native microbiomes. Several photosynthetic taxa in the leaves (e.g. *Cyanobacteria*, *Chromatiales*) were negatively related to the photosynthetic efficiency of the plants (Fv/Fm), which suggests that some microbial taxa of the phyllosphere might be negatively impacting the plant cells.

While seasonal succession has been widely demonstrated for the microbial community in the phyllosphere of terrestrial plants [31], only one study reported seasonal changes in the microbial community of leaves in the seagrass *Cymodocea nodosa* and the macroalga *Caulerpa cylindracea* [42]. This last study did not repeat any season, and therefore could not verify the recurrence of the seasonal dynamics. Interestingly, our results suggest, to our knowledge for the first time, a recurrent pattern in the leaf microbiome of *Zostera marina.* Intriguingly, the succession seems to be disrupted in Cesantes, concurring with a drop in richness and diversity, likely reflecting a strong alteration in the meadow status. Even though the temporal variability of root microbiome was lower than that in leaves, the potential disruption in Cesantes could be also appreciated in the roots. Nevertheless, it is important to note that more frequent and multiyear samplings would be necessary to corroborate the existence of a recurrent seasonal pattern in the plant microbiomes.

The possible disruption of the succession pattern, as well as the overall lower richness, diversity, and equitability of root microbiome in Cesantes compared to Bouzas in March 2022 might be related to the higher amount of macroalgae biomass in the former meadow, which was ca. fourfold higher in Cesantes than in Bouzas. The supplementary input of organic matter associated to macroalgae growth might subsequently fuel the proliferation of fast-growing copiotrophic microbes, altering microbial community composition, and reducing the diversity. An inverse relationship was indeed observed between the richness in roots and the amount of organic matter in the sediments. A reduction of bacterial diversity associated to the selection of copiotrophic taxa, has been consistently reported in soils ([46] and references therein). Even though the Cesantes beach is expected to be less anthropized than Bouzas beach, nutrient inputs associated to riverine and continental groundwater discharge are higher in Cesantes than in Bouzas [35]. The higher nutrient inputs together with the low energy hydrodynamic conditions in Cesantes, located in the inner part of the ría [4], increase the water retention time and favour macroalgae proliferation and accumulation compared to Bouzas beach. Episodic and extense blooms of green seaweeds of the *Ulva* genus, which may cause severe impacts on benthic macrofauna and shell fishing, recurrently occur in the inner part of the ria [29, 55]. While the seagrass detritus is relatively refractory due to the high lignocellulosic compounds, the detritus of macroalgae are more labile and may enhance microbial decomposition [47], creating conditions favourable for nitrogen fixation or sulfate reduction (e.g. anoxic conditions). Several studies reported higher N fixation rates in the *Z. marina* rhizosphere than in adjacent bare sediments and suggested that these rates were mainly sustained by epiphytic heterotrophic bacteria and were strongly related to sediment organic content [10, 11].

The hypothesis of more anoxic conditions and intensified N and S cycles in Cesantes than in Bouzas is consistent with the higher sediment organic content and with the microbiome composition of roots. The anaerobic *Modulibacteria*, which have been identified as indicators of high temperature and low light conditions in seagrass meadows [76], were more abundant in Cesantes than in Bouzas. *Desulfobacterota* also showed higher relative abundance in the root microbiome of the Cesantes meadow, compared with Bouzas. These sulfate-reducing bacteria appear to be the main nitrogen fixers in roots of seagrasses, providing a large fraction of the plant nitrogen requirements [69, 82]. The relative abundance of *Campylobacterota* and *Crenarchaeota* is also higher in Cesantes than in Bouzas. *Campylobacterota*, which may also contribute to nitrogen fixation [79], are capable of sulfide oxidization using nitrate as electron donor [71], while the *Crenarchaeota* play a major role in ammonia oxidation [84]. The nitrite oxidizers *Chloroflexi* [70] were similarly abundant in the roots of both meadows. A higher nitrogen fixation in Cesantes compared to Bouzas could explain the higher N content of the rhizomes, regardless of the similar DIN concentration found in sediment porewaters at the two sites. Accordingly, Cole and McGlathery [11] estimated that nitrogen fixation in *Z. marina* meadows significantly contribute to the N demand of the plant.

## Conclusions

A close connection was found between microbial taxonomic composition of leaves and roots and seagrass characteristics, suggesting a role of root microbiome on the plant growth. A tight coupling between nitrogen and sulfur cycles is evident from the root microbiome composition of the *Z. marina* meadows, with taxa involved in the transformations of these elements being more dominant in Cesantes, likely as a result of a high input of macroalgae biomass. The exploration of the *Z. marina* microbiome in leaves and roots in these two nearby locations differently affected by anthropogenic pressures revealed differences in taxonomic composition which strongly supports the utility of microbial taxa as early indicators of seagrasses ecological status [12, 50]. Further multiyear studies would allow defining recurrent seasonal succession patters, and would set the basis for monitoring seasonal changes, as disruptions of the succession patterns may better reflect alterations in the meadows than the abundance of particular taxa.

## Supplementary Information

Additional file1 (PDF 983 KB)

Additional file2 (PDF 194 KB)

Additional file3 (PDF 117 KB)

Additional file4 (PDF 156 KB)

Additional file5 (PDF 75 KB)

Additional file6 (DOCX 17 KB)

## Data Availability

The sequence data from this study have been deposited at the NCBI GenBank database under accession number PRJNA1210619 (https://www.ncbi.nlm.nih.gov/bioproject/1210619).
