# Supplementary material for: Contrasting diversity and temporal patterns in leaf and root microbiome of two nearby temperate Zostera marina meadows
Source: Environ Microbiome. 2025 Aug 5;20:98. doi: 10.1186/s40793-025-00760-z (PMC12326708; doi:10.1186/s40793-025-00760-z)
Supplement: Supplementary file 1 — Additional file1 (PDF 983 KB) [file 40793_2025_760_MOESM1_ESM.pdf]

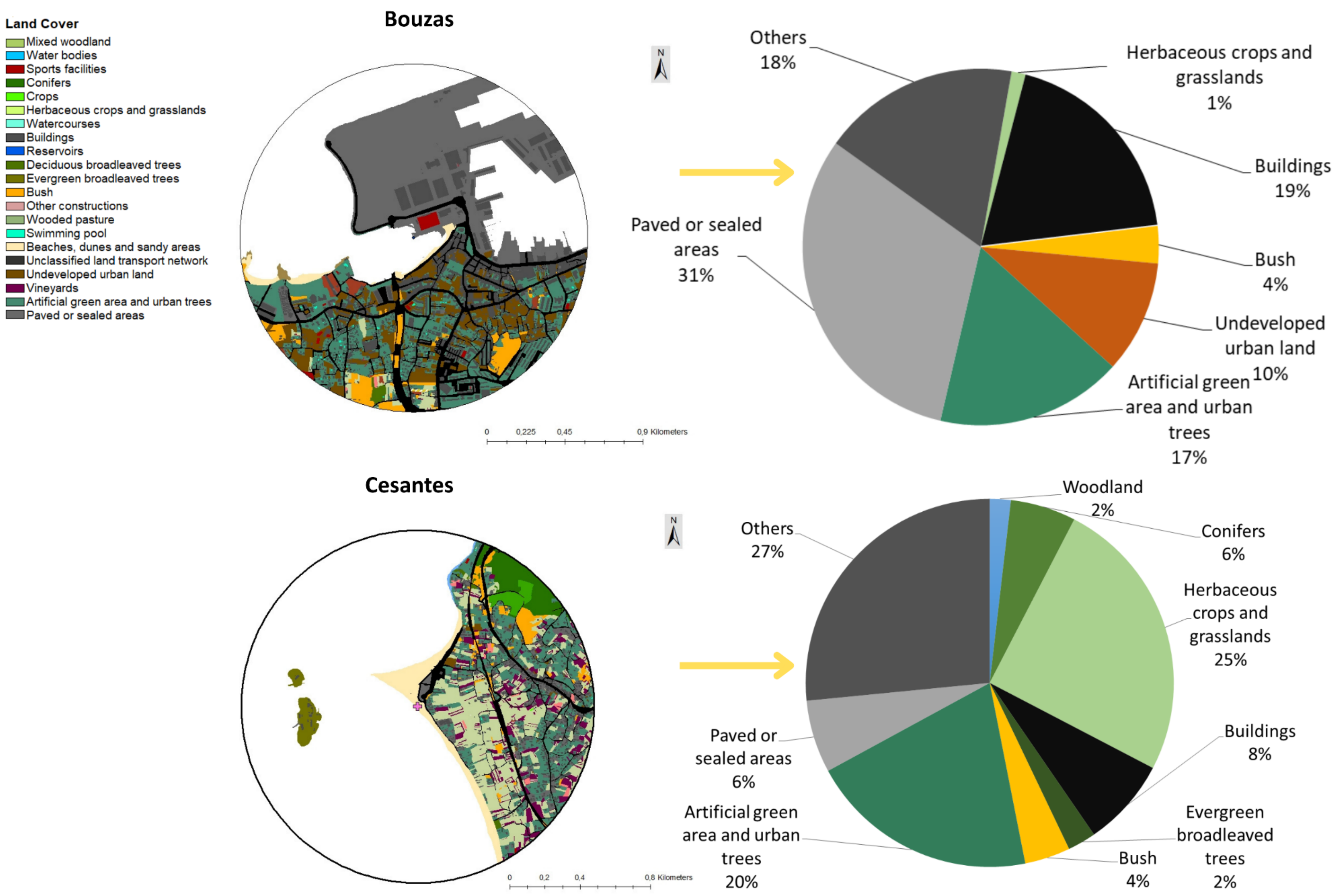

Figure S1. Spatial distribution of land cover classes in the surroundings (1000 m radius) of selected sampling stations (Bouzas and Cesantes) and the corresponding relative contribution of each class to the total terrestrial area. Land cover data obtained from the most recent (2004) SIOSE Land Use Information System.
