# Supplementary material for: Contrasting diversity and temporal patterns in leaf and root microbiome of two nearby temperate Zostera marina meadows
Source: Environ Microbiome. 2025 Aug 5;20:98. doi: 10.1186/s40793-025-00760-z (PMC12326708; doi:10.1186/s40793-025-00760-z)
Supplement: Supplementary file 2 — Additional file2 (PDF 194 KB) [file 40793_2025_760_MOESM2_ESM.pdf]

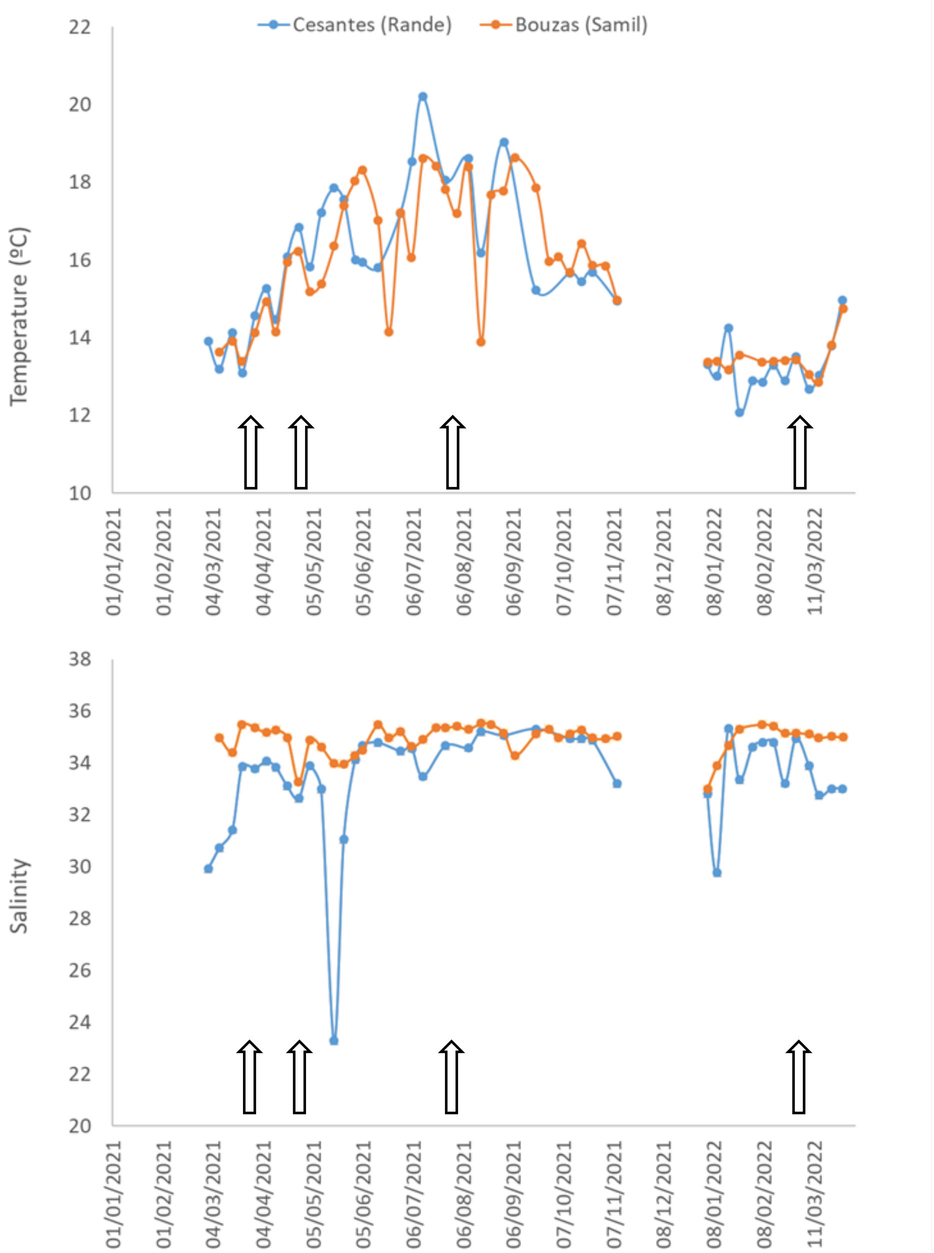

Figure S2. Plots showing weekly collected temperature and salinity at two marine stations located close to the Bouzas and Cesantes meadows. The arrows indicate the data of sampling at the meadows. Data obtained from INTECMAR (https://www.intecmar.gal/Ctd/Default.aspx).
