# Supplementary material for: Contrasting diversity and temporal patterns in leaf and root microbiome of two nearby temperate Zostera marina meadows
Source: Environ Microbiome. 2025 Aug 5;20:98. doi: 10.1186/s40793-025-00760-z (PMC12326708; doi:10.1186/s40793-025-00760-z)
Supplement: Supplementary file 3 — Additional file3 (PDF 117 KB) [file 40793_2025_760_MOESM3_ESM.pdf]

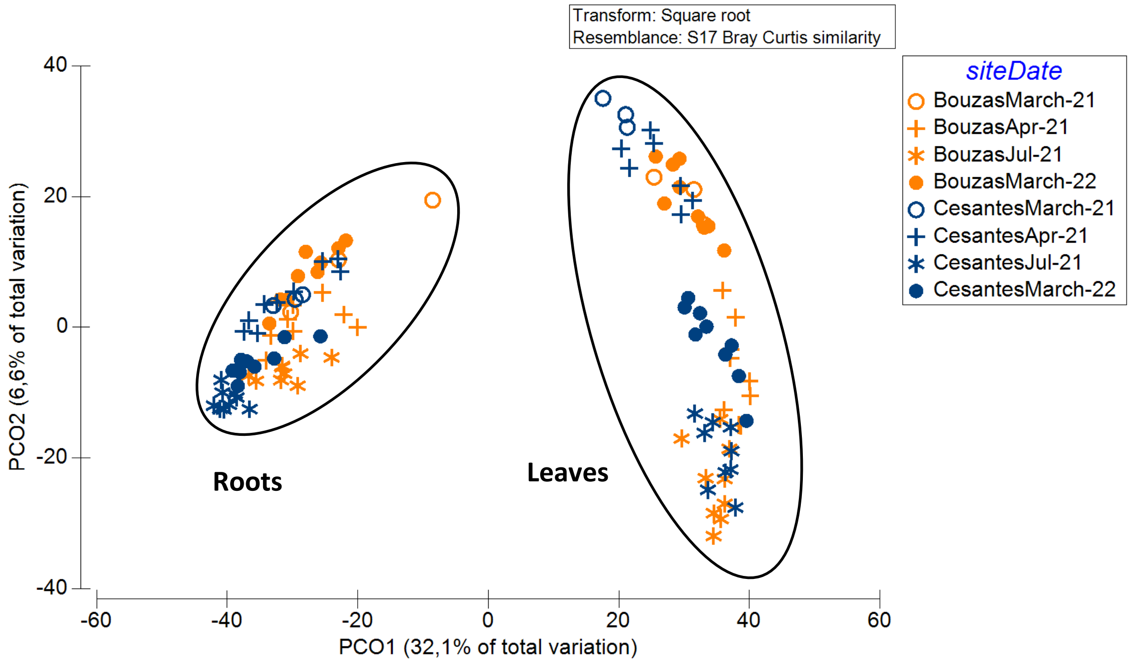

Figure S3. Principal coordinate analysis showing ordination of all the samples included in this study.
