# Supplementary material for: Contrasting diversity and temporal patterns in leaf and root microbiome of two nearby temperate Zostera marina meadows
Source: Environ Microbiome. 2025 Aug 5;20:98. doi: 10.1186/s40793-025-00760-z (PMC12326708; doi:10.1186/s40793-025-00760-z)
Supplement: Supplementary file 4 — Additional file4 (PDF 156 KB) [file 40793_2025_760_MOESM4_ESM.pdf]

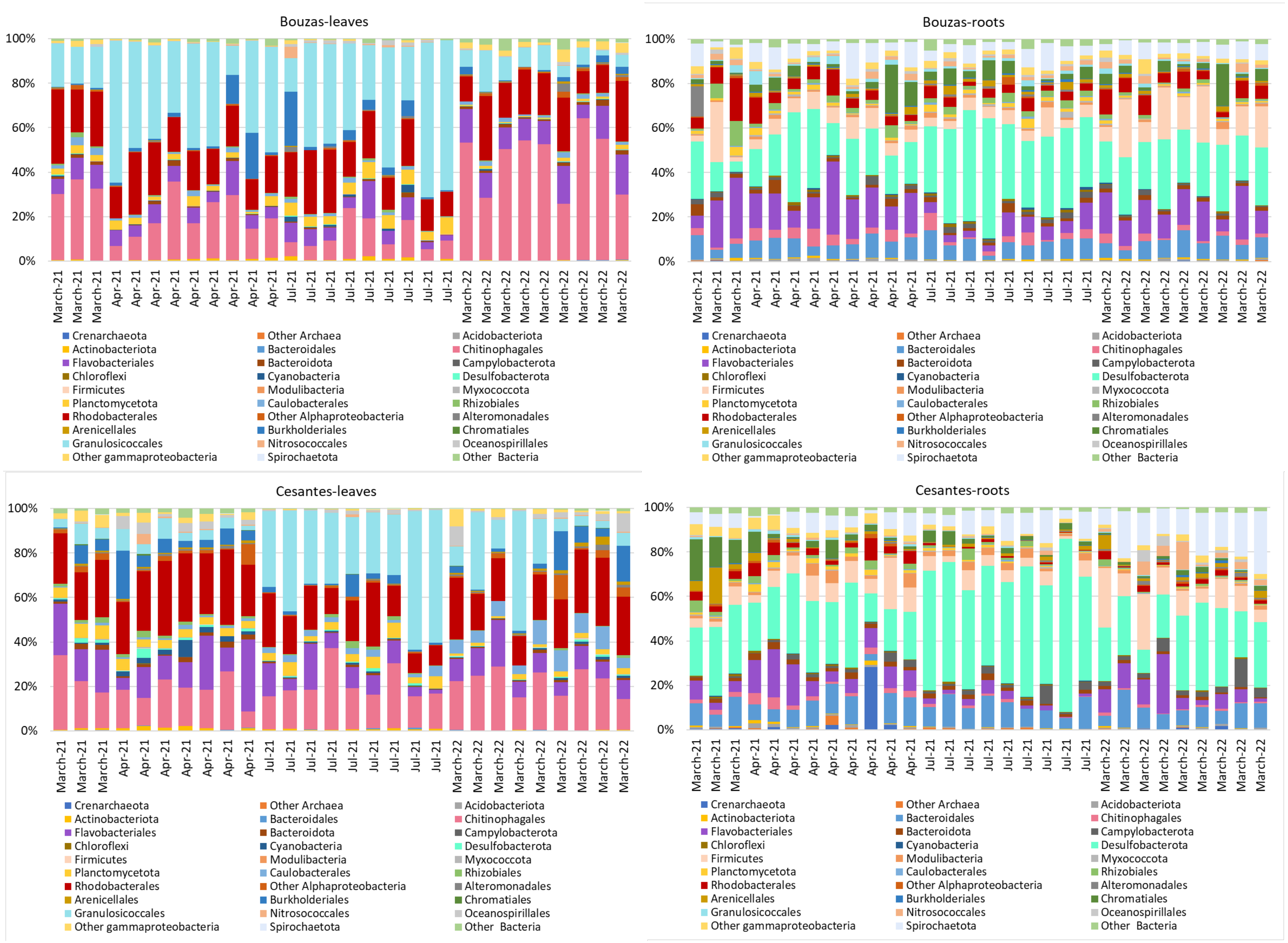

Figure S4. Relative contribution of major prokaryote taxa to leaf and root microbiomes in Bouzas and Cesantes.
