# Supplementary material for: Contrasting diversity and temporal patterns in leaf and root microbiome of two nearby temperate Zostera marina meadows
Source: Environ Microbiome. 2025 Aug 5;20:98. doi: 10.1186/s40793-025-00760-z (PMC12326708; doi:10.1186/s40793-025-00760-z)
Supplement: Supplementary file 5 — Additional file5 (PDF 75 KB) [file 40793_2025_760_MOESM5_ESM.pdf]

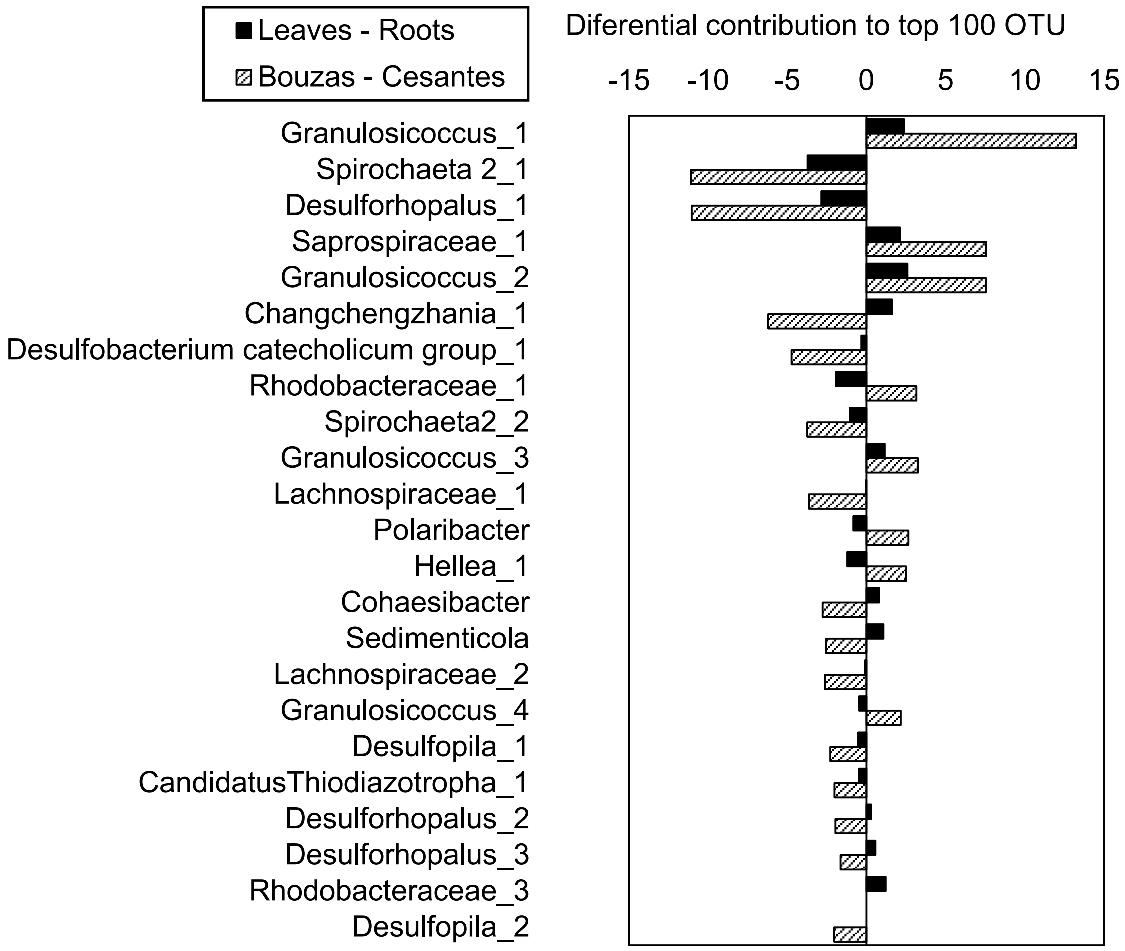

Figure S5. Plot showing the differential contribution (in %) to the top 100 OTU abundance in leaves vs. roots (black bar) and in Bouzas vs Cesantes (patterned bar) of the most significant OTUs obtained in the SIMPER analysis. Negative values represent OTUs more abundant in roots or in Cesantes while positive values correspond to OTUs more abundant in leaves or in Bouzas.
