# Supplementary material for: Contrasting diversity and temporal patterns in leaf and root microbiome of two nearby temperate Zostera marina meadows
Source: Environ Microbiome. 2025 Aug 5;20:98. doi: 10.1186/s40793-025-00760-z (PMC12326708; doi:10.1186/s40793-025-00760-z)
Supplement: Supplementary file 6 — Additional file6 (DOCX 17 KB) [file 40793_2025_760_MOESM6_ESM.docx]

Table S1. Mean density, growth, relative growth, algae biomass and necrosis in the two location for each of the sampling events. ND, not determined. SD, standard deviation from 3 replicates. March-21 was the time zero for growth estimates.

|  |  |  | Density | Growth | Relative growth | | Necrosis | |
| --- | --- | --- | --- | --- | --- | --- | --- | --- |
| Location | Date |  | shoots m^-2^ | cm shoot^-1^ d^-1^ | | d^-1^ | | cm shoot^-1^ |
| Bouzas | March-21 | **Mean** | **172,4** | *ND* | | *ND* | | **2,8** |
|  |  | *SD* | *6,7* | *ND* | | *ND* | | 1,2 |
|  | Apr-21 | **Mean** | **223,4** | **3,8** | | **67,1** | | **1,8** |
|  |  | *SD* | *30,3* | *0,7* | | *7,3* | | 0,6 |
|  | Jul-21 | **Mean** | **340,7** | **4,9** | | **78,3** | | *ND* |
|  |  | *SD* | *52,9* | *0,8* | | *4,7* | | *ND* |
|  | March-22 | **Mean** | **184,6** | **3,3** | | **64,3** | | **1,6** |
|  |  | *SD* | *49,3* | *0,6* | | *8,4* | | 0,5 |
| Cesantes | March-21 | **Mean** | **156,4** | *ND* | | *ND* | | **17,3** |
|  |  | *SD* | *11,0* | *ND* | | *ND* | | 7,4 |
|  | Apr-21 | **Mean** | **150,5** | **4,0** | | **58,5** | | **1,3** |
|  |  | *SD* | *20,9* | *0,5* | | *6,1* | | 0,4 |
|  | Jul-21 | **Mean** | **234,1** | **5,2** | | **79,1** | | *ND* |
|  |  | *SD* | *27,5* | *0,7* | | *6,7* | | *ND* |
|  | March-22 | **Mean** | **256,2** | **3,6** | | **65,2** | | **3,4** |
|  |  | *SD* | *65,5* | *1,0* | | *16,1* | | *1,5* |

Table S2. C, N and C/N content in leaves and rhizomes in Bouzas and Cesantes. Content expressed as %. The mean for each sampling location is provided. SD, standard deviation.

| Location | Date | Leaves |  |  | Rhizome |  |  |
| --- | --- | --- | --- | --- | --- | --- | --- |
|  |  | % C | % N | C/N | % C | % N | C/N |
| Bouzas | March-21 | 36,8 | 2,1 | 20,9 | 32,9 | 0,8 | 52,2 |
|  | Apr-21 | 37,0 | 2,1 | 20,4 | 27,9 | 0,9 | 39,0 |
|  | Apr-21 | 40,1 | 2,1 | 22,0 | 25,5 | 0,6 | 54,8 |
|  | Jul-21 | 35,8 | 1,8 | 23,8 | 28,2 | 0,7 | 46,8 |
|  | Jul-21 | 38,6 | 1,2 | 36,1 | 55,7 | 0,7 | 98,1 |
|  | March-22 | 34,4 | 2,3 | 17,5 | 29,7 | 0,7 | 52,8 |
|  | March-22 | 31,5 | 2,2 | 16,8 | 30,5 | 0,5 | 76,8 |
|  | **Mean** | **36,3** | **2,0** | **22,5** | **32,9** | **0,7** | **60,1** |
|  | *SD* | *2,6* | *0,3* | *6,0* | *9,5* | *0,1* | *18,8* |
| Cesantes | March-21 | 37,2 | 2,6 | 16,7 | 29,4 | 1,3 | 27,7 |
|  | Apr-21 | 40,2 | 2,2 | 21,4 | 28,9 | 1,4 | 25,1 |
|  | Apr-21 | 38,3 | 1,9 | 23,6 | 27,7 | 0,8 | 41,1 |
|  | Jul-21 | 35,2 | 1,5 | 27,7 | 31,5 | 1,2 | 30,5 |
|  | Jul-21 | 37,5 | 1,9 | 23,8 | 34,1 | 0,9 | 44,7 |
|  | March-22 | 27,8 | 2,1 | 15,4 | 34,4 | 1,4 | 27,8 |
|  | March-22 | 30,6 | 2,3 | 15,9 | 33,0 | 1,3 | 30,0 |
|  | **Mean** | **35,3** | **2,1** | **20,6** | **31,3** | **1,2** | **32,4** |
|  | *SD* | *4,1* | *0,3* | *4,4* | *2,5* | *0,2* | *6,9* |
